# Insulin resistance according to β-cell function in women with polycystic ovary syndrome and normal glucose tolerance

**DOI:** 10.1371/journal.pone.0178120

**Published:** 2017-05-25

**Authors:** Do Kyeong Song, Young Sun Hong, Yeon-Ah Sung, Hyejin Lee

**Affiliations:** Department of Internal Medicine, Ewha Womans University School of Medicine, Seoul, Korea; John Hopkins University School of Medicine, UNITED STATES

## Abstract

**Background:**

Polycystic ovary syndrome (PCOS) is associated with insulin resistance (IR) and compensatory hyperinsulinemia. IR is recognized as a major risk factor for the development of type 2 diabetes mellitus. However, few studies have investigated IR in women with PCOS and normal glucose tolerance. The objective of this study was to evaluate IR and β-cell function in women with PCOS and normal glucose tolerance. Additionally, we sought to evaluate the usefulness of oral glucose tolerance test (OGTT)-derived IR indices in lean women with PCOS.

**Methods:**

We recruited 100 women with PCOS and normal glucose tolerance and 100 age- and BMI-matched women as controls. IR and insulin secretory indices, including the homeostasis-model assessment (HOMA)-IR, HOMA-M_120_, HOMA-F and the Stumvoll index, were calculated from an OGTT. Increased β-cell function was defined as>75th percentile for the HOMA-F in control women.

**Results:**

Women with PCOS had higher values for post-load 2-hour glucose, fasting insulin, post-load 2-hour insulin, HOMA-IR, HOMA-M_120_, HOMA-F and lower values for the Stumvoll index than the controls (all *P*s<0.05). Women with PCOS and increased β-cell function showed lower Stumvoll index values than the matched controls (*P*<0.05). The HOMA-F was significantly associated with the HOMA-M_120_ and Stumvoll index when adjusted for age and BMI in a multiple regression analysis (all *P*s<0.05). The HOMA-M_120_ was positively correlated with triglycerides and free testosterone, and the Stumvoll index was negatively correlated with triglycerides and free testosterone in lean women with PCOS (all *P*s<0.05).

**Conclusions:**

Women with PCOS and normal glucose tolerance showed higher IR than controls matched for age, BMI, and β-cell function. β-cell function was increased in women with PCOS when compared to the matched controls, but not when the lean subjects were compared to the matched controls separately. Therefore, early evaluation of IR in women with PCOS and normal glucose tolerance may be needed.

## Introduction

Polycystic ovary syndrome (PCOS) is the most common endocrine disorder in women of reproductive age and is characterized by ovulatory dysfunction, hyperandrogenism, and polycystic ovary morphology as well as metabolic abnormalities, including hypertension and dyslipidemia [1]. PCOS is also associated with insulin resistance (IR), and approximately 50% to 70% of women with PCOS have IR. IR is recognized as a major risk factor for the development of type 2 diabetes mellitus (T2DM), which is 5-to 10-fold higher in women with PCOS than in healthy controls [2]. A previous study showed that Chinese women with PCOS and normal glucose tolerance had higher IR than controls, even in lean subjects [3]. However, few studies have investigated IR in women with PCOS and normal glucose tolerance, especially in lean women with PCOS. IR evaluation in lean women with PCOS could be underestimated owing to the absence of obesity. Recently, the homeostasis-model assessment (HOMA)-M_120_ was reported as a simple and reliable measure of IR for lean European women with PCOS [4]. However, the efficacy of HOMA-M_120_ for estimating IR in lean women with PCOS has not been evaluated in an Asian population.

When insulin secretion becomes inadequate in response to IR, impaired glucose tolerance (IGT) and T2DM develop. However, studies of β-cell function in women with PCOS have provided controversial results. Women with PCOS showed greater insulin secretion to maintain normal glucose homeostasis than age and body mass index (BMI)-matched controls in a European population [5]. However, early impaired β-cell function was detected in Chinese women with PCOS [6]. The objectives of this study were to evaluate IR and β-cell function in women with PCOS and normal glucose tolerance, and to evaluate the usefulness of IR indices in lean Asian women with PCOS using an oral glucose tolerance test (OGTT).

## Materials and methods

### Subjects

Between 2008 and 2010, we performed a survey of the health and nutritional status of women under 40 years of age who were residents of Seoul, Korea. Of 2,950 women who were recruited by newspaper and online advertisements and voluntarily participated in this survey, we conducted the ultrasound scans and OGTT in 550 women who agreed the examinations. Among them, subjects were excluded if they had been on any medication within 3 months of the evaluation or had used other drugs that could affect sex hormone metabolism or insulin action. Finally, we enrolled 100 women with PCOS (aged 16–36 yr; 50 lean with mean BMI 20.4 ± 1.6 kg/m^2^ and 50 overweight/obese with mean BMI 25.2 ± 2.3 kg/m^2^). We also included 100 age- and BMI-matched women with normal glucose tolerance as controls. All women completed a standard OGTT with measurement of glucose and insulin levels at 30 min intervals. All women had normal fasting glucose (< 100 mg/dL) and glucose tolerance (< 140 mg/dL at 2 h).

As proposed by the European Society for Human Reproduction and Embryology [7], PCOS was diagnosed when two or more of the following three criteria were met: oligo- or anovulation, hyperandrogenism, or polycystic ovaries. Oligomenorrhea was defined as fewer than ten menstrual cycles per year. Biochemical hyperandrogenemia was defined as a total or free testosterone level above the 95^th^ percentile (total testosterone ≥ 67 ng/dL or free testosterone ≥ 0.84 ng/dL) of the testosterone levels of healthy, regular-cycling women [8]. Clinical hyperandrogenism was defined as the presence of hirsutism with a modified Ferriman-Gallwey score ≥ 3 [9]. The criteria for the diagnosis of polycystic ovaries required the visualization of ≥ 12 follicles/ovary that were 2 to 9 mm in diameter or an ovarian volume >10 cm^3^ by transvaginal ultrasonography.

Patients with similar clinical presentations, such as congenital adrenal hyperplasia, androgen-secreting tumors, and Cushing’s syndrome, were excluded [7]. Patients with 21-hydroxylase-deficient non-classical adrenal hyperplasia were excluded using a 17-hydroxyprogesterone level cut-off of > 2 ng/mL. In all cases and controls, overweight was defined as BMI ≥ 23 kg/m^2^ and obesity as a BMI ≥ 25 kg/m^2^ according to the BMI cut-off points of obesity for Asian populations [10]. Written informed consent was obtained from all of the participants, and the institutional review board of Ewha Womans University Mokdong Hospital approved this study.

### Methods

Height and weight were measured for all subjects, and BMI was calculated as weight (kg)/height (m)^2^. Waist circumference (WC) was measured in a standing position midway between the lower costal margin and the iliac crest. Blood pressure was calculated as the mean of two manual sphygmomanometer readings with the patient in a seated position. A single-frequency bioelectrical impedance plethysmograph was used for estimating body fat mass (In Body 230, Biospace Industry, Seoul, Korea). Standard electrocardiographic electrodes were placed on the hands and feet of the subjects, and isopropyl alcohol was used to clean each electrode attachment site. The subject’s legs were parted, and the arms were adducted by approximately 30° to prevent skin-to-skin contact. The percentage of fat was defined as the total mass of fat divided by the total body mass.

On the third day of the follicular phase of the menstrual cycle, a venous blood sample was obtained from each subject after an overnight fast of at least 8 h. In women with amenorrhea, the blood samples were obtained on a random day. Total testosterone levels were measured via the chemiluminescent immunoassay method using a commercially available kit (Siemens, NY, USA; mean inter- and intra-assay CV were 4.4% and 6.2%, respectively). Sex hormone-binding globulin (SHBG) levels were measured by immunoradiometric assay using a commercially available kit (DPC, Los Angeles, CA, USA; mean inter- and intra-assay CV were 7.9% and 5.3%, respectively). Free testosterone levels were calculated using the formula available from the International Society for Study of the Aging Male website, which is based on total testosterone, SHBG, and albumin levels in the same sample from each subject. The free androgen index (FAI) was calculated as testosterone (in nanomoles per liter)/SHBG (in nanomoles per liter) × 100.

Ultrasound examinations were performed with a 7MHz transvaginal (or transabdominal for virginal women) transducer (Logic 400 General Electric, Milwaukee, WI, USA). Ovarian volume was calculated according to a simplified formula for an ellipsoid (0.5 × length × width × thickness) [11]. Ovarian volume was defined as the average volume of both ovaries, and the ovarian follicle number as the average number of follicles in each ovary.

The 75-g OGTT was performed in the morning after an overnight fast. After 30 min of supine rest, venous blood samples were drawn at baseline, 90 min, and 120 min after the 75-g glucose load. Plasma glucose levels were measured via the glucose oxidase method (Beckman Model Glucose Analyzer 2, CA, USA), and insulin levels were measured by radioimmunoassay using a commercially available kit (BioSource, Nivelles, Belgium). Fasting total serum cholesterol, triglycerides (TG), and high-density lipoprotein (HDL) cholesterol levels were measured using an enzymatic assay on an automated analyzer (Hitachi 7150 Automatic Chemistry Analyzer, Japan). β-cell function was assessed by the homeostasis model assessment of β-cell function (HOMA-F): 20 × fasting insulin, mIU/L/(fasting glucose, mmol/L—3.5). Increased β-cell function was defined as > 75^th^ percentile of HOMA-F, which was 122.44 mIU/ in 100 regular cycling healthy women. OGTT-derived insulin sensitivity indices including the HOMA-IR, M_120_ and Stumvoll index [0.226−(0.0032 × BMI)—(0.0000645 × I_120,_ pmol/L)—(0.00375 × G_90,_ mmol/L)] were calculated from OGTT. The HOMA-IR was calculated as the product of the fasting insulin level (mIU/L) and the fasting glucose level (mg/dL) divided by 405. The modified HOMA-M_y_ indices were calculated as follows: HOMA-M_y_ = G_y_ (mg/dL) × I_y_ (mIU/L)/405, where _y_ and I_y_ indicate 120-minute glucose and insulin values from the OGTT, respectively [4].

### Statistical analyses

Statistical analyses were performed using the SPSS 18.0 software package for Windows (IBM Corporation, Chicago, IL, USA). The Kolmogorov-Smirnov statistic was used to analyze the continuous variables for normality. The levels of the HOMA-IR, HOMA-M_120_, Stumvoll index, and HOMA-F were logarithmically transformed to achieve a normal distribution. Quantitative variables were reported as the mean ± standard deviation. Variables that showed a skewed deviation were reported with medians and interquartile ranges. Between group differences were assessed by the unpaired *t* test or Mann-Whitney U-test. The Spearman’s rho correlation coefficient was applied to assess the correlation between the insulin sensitivity indices and the hormonal and metabolic features of PCOS. Multiple linear regression analyses were performed to determine the independent association between HOMA-F and the insulin resistance indices. A *P* value < 0.05 was considered significant.

## Results

Women with PCOS had higher values for post-load 2-hour glucose, fasting insulin, post-load 2-hour insulin, HOMA-IR, HOMA-M_120_, HOMA-F and lower value of Stumvoll index values than the corresponding controls (all *P*s < 0.05) (Table 1). Lean women with PCOS had higher values for post-load 2-hour insulin and HOMA-M_120_ and lower value of Stumvoll index values than the corresponding controls (all *P*s < 0.05) (Table 2).

**Table 1:** Clinical and biochemical characteristics.

	PCOS (n = 100)	Control (n = 100)	*P*-value
**Age (years)**	23 ± 5	23 ± 4	0.961
**Body mass index (kg/m**^**2**^**)**	22.8 ± 3.1	22.7 ± 3.0	0.919
**Percentage of fat (%)**	34.2 ± 5.6	34.5 ± 5.2	0.572
**Fat mass (kg)**	20.5 ± 5.6	21.2 ± 5.8	0.758
**Sex hormone-binding globulin (nmol/l)**	51.6 ± 22.2	89.8 ± 44.3	<0.001
**Total testosterone (ng/dl)**	80.7 ± 17.8	44.3 ± 13.7	0.024
**Free testosterone (ng/dl)**	1.15 ± 0.35	0.43 ± 0.19	<0.001
**Free androgen index**	6.4 ± 3.2	2.1 ± 1.1	<0.001
**Fasting glucose (mg/dL)**	84 ± 7	84 ± 7	0.938
**Post-load 2-h glucose (mg/dL)**	98 ± 16	93 ± 18	0.031
**Fasting insulin (mIU/L)**	6.61 (2.06, 9.28)	3.84 (0.30, 7.20)	0.002
**Post-load 2-h insulin (mIU/L)**	31.81 (16.93, 62.76)	22.63 (12.98, 36.81)	0.002
**HOMA-IR**	1.27 (0.42, 1.95)	0.75 (0.07, 1.50)	0.004
**HOMA-M**_**120**_	7.47 (3.68, 15.61)	5.11 (2.51, 8.70)	0.001
**ISI Stumvoll (μmol/kg. min). (pmol/L)**^**-1**^	0.10 (0.09, 0.11)	0.11 (0.10, 0.12)	<0.001
**HOMA-F (mIU/mmol)**	116.91 (26.41, 173.45)	68.70 (3.87, 122.44)	0.003
**Ovarian volume (cm**^**3**^**)**	8.4 ± 3.6	4.7 ± 1.5	<0.001
**Ovarian follicle no.**	10.0 ± 6.3	6.0 ± 1.7	0.010

Plus-minus values are mean ± SD.

Values before parentheses are medians, and values in parentheses are interquartile ranges. PCOS, polycystic ovary syndrome; HOMA-IR, homeostasis model assessment of insulin resistance; ISI, insulin sensitivity index; HOMA-F, homeostasis model assessment β-cell function.

**Table 2:** Clinical and biochemical characteristics in lean subjects.

	PCOS (n = 50)	Control (n = 50)	*P*-value
**Age (years)**	23 ± 5	22 ± 4	0.185
**Body mass index (kg/m**^**2**^**)**	20.4 ± 1.6	20.5 ± 1.8	0.785
**Percentage of fat (%)**	30.6 ± 4.4	31.1 ± 3.9	0.576
**Fat mass (kg)**	16.4 ± 3.1	16.9 ± 3.2	0.759
**Sex hormone-binding globulin (nmol/l)**	59.9 ± 20.2	101.8 ± 42.8	<0.001
**Total testosterone (ng/dl)**	84.7 ± 17.2	43.2 ± 13.8	0.094
**Free testosterone (ng/dl)**	1.07 ± 0.32	0.37 ± 0.16	<0.001
**Free androgen index**	5.5 ± 2.1	1.7 ± 0.8	<0.001
**Fasting glucose (mg/dL)**	84 ± 7	84 ± 6	0.951
**Post-load 2-h glucose (mg/dL)**	94 ± 12	91 ± 18	0.311
**Fasting insulin (mIU/L)**	5.70 (1.81–8.03)	3.41 (0.14–6.75)	0.066
**Post-load 2-h insulin (mIU/L)**	28.65 (17.71–47.25)	19.82 (11.06–30.46)	0.007
**HOMA-IR**	1.17 (0.38–1.58)	0.74 (0.03–1.36)	0.091
**HOMA-M**_**120**_	6.95 (3.60–10.73)	4.44 (2.31–7.29)	0.005
**ISI-Stumvoll (μmol/kg. min). (pmol/L)**^**-1**^	0.106 (0.095–0.114)	0.113 (0.103–0.123)	0.010
**HOMA-F (mIU/mmol)**	95.34 (21.09–148.99)	68.70 (2.07–121.70)	0.097
**Ovarian volume (cm**^**3**^**)**	9.4 ± 3.6	4.6 ± 1.4	<0.001
**Ovarian follicle no.**	9.7 ± 2.7	6.1 ± 1.7	0.001

Plus-minus values are mean ± SD.

Values before parentheses are medians, and values in parentheses are interquartile ranges. PCOS, polycystic ovary syndrome; HOMA-IR, homeostasis model assessment of insulin resistance; ISI, insulin sensitivity index; HOMA-F, homeostasis model assessment β-cell function.

**Fig 1:**
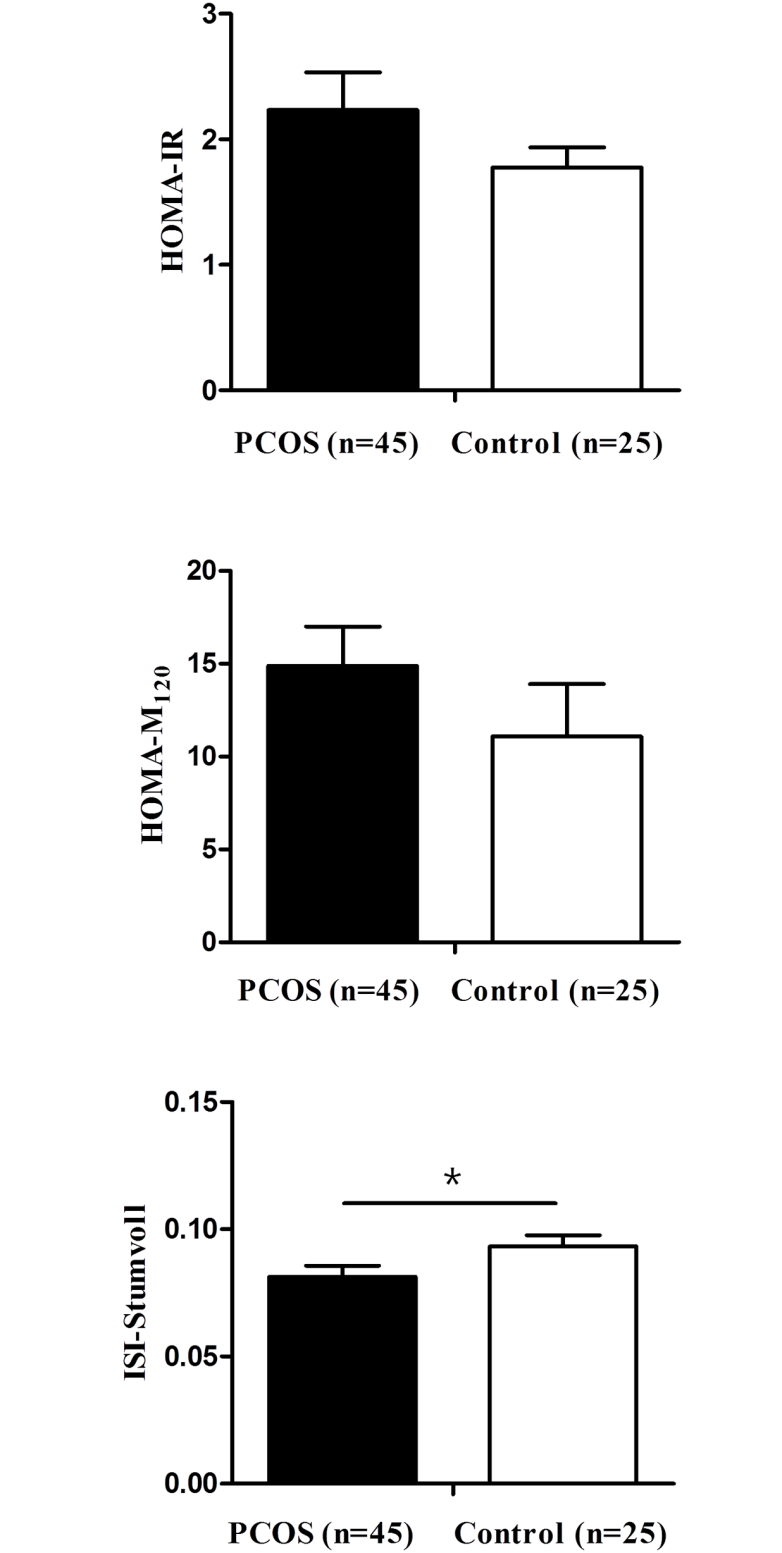
Comparison of insulin resistance indices in women with increased β-cell function. * *P* < 0.05 vs. controls. PCOS, polycystic ovary syndrome; HOMA-IR, homeostasis model assessment of insulin resistance; ISI, insulin sensitivity index.

**Fig 2:**
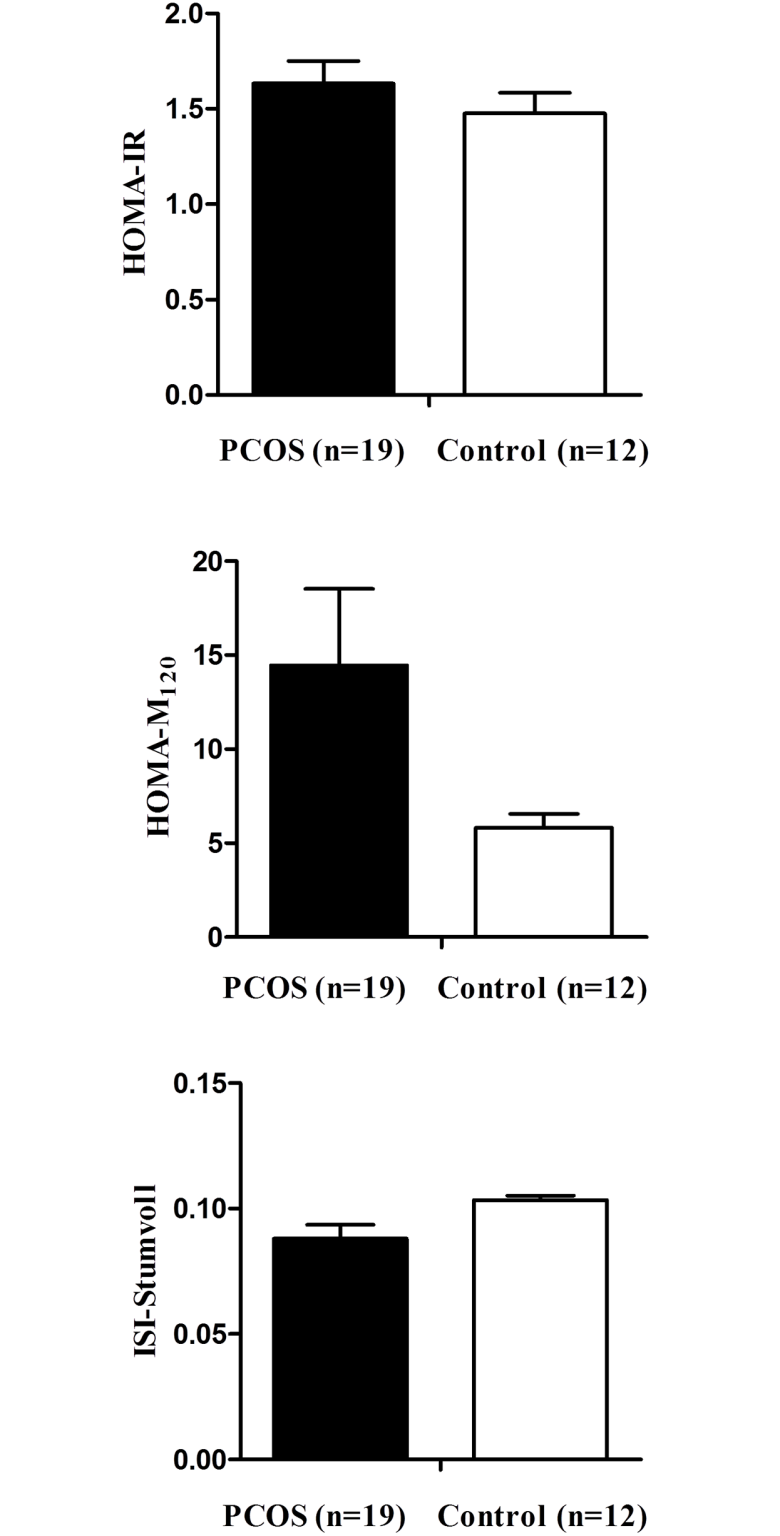
Comparison of insulin resistance indices in lean women with increased β-cell function. * *P* < 0.05 vs. controls. PCOS, polycystic ovary syndrome; HOMA-IR, homeostasis model assessment of insulin resistance; ISI, insulin sensitivity index.

Women with PCOS and increased β-cell function showed lower value of Stumvoll index values than controls with increased β-cell function (*P* < 0.05) (Fig 1). The HOMA-F did not differ between women with PCOS and controls with increased β-cell function (*P* = 0.120, data not shown). Lean women with PCOS and increased β-cell function showed a higher value for HOMA-M_120_ and lower value of Stumvoll index values than lean controls with increased β-cell function (all *P*s < 0.05) (Fig 2).

In all women with PCOS, multiple linear regression analysis showed that the HOMA-F was positively associated with the HOMA-M_120_ (β = 0.321) and negatively associated with the Stumvoll index (β = -0.287) when adjusted for age and BMI (all *P*s < 0.05). In lean women with PCOS, the HOMA-F was positively associated with the HOMA-M_120_ (β = 0.272) at a marginally significant level when adjusted for age and BMI (Table 3).

**Table 3:** Multiple linear regression analyses of HOMA-F for insulin resistance indices.

Dependent variables	Unstandardized coefficients		Standardized coefficients	*P*-value	C.I.
	B	S.E.	beta		
**Total PCOS (n = 100)**					
**HOMA-M**_**120**_	0.176	0.053	0.321	0.001	0.070 ~ 0.282
**ISI Stumvoll**	- 0.073	0.026	- 0.287	0.006	- 0.125 ~ -0.022
**Lean PCOS (n = 50)**					
**HOMA-M**_**120**_	0.168	0.087	0.272	0.062	- 0.009 ~ 0.344
**ISI Stumvoll**	- 0.087	0.053	- 0.237	0.112	- 0.194 ~ 0.021

Adjusted by age and body mass index.

HOMA-F, HOMA-IR, HOMA-M_120_, and ISI Stumvoll were logarithmically transformed

HOMA-F, homeostasis model assessment β-cell function; HOMA-IR, homeostasis model assessment of insulin resistance; ISI, insulin sensitivity index.

In women with PCOS, the HOMA-IR and HOMA-M_120_ were positively correlated with WC, TG, free testosterone, and FAI and negatively correlated with HDL cholesterol and SHBG. The Stumvoll index was positively correlated with HDL cholesterol and SHBG and negatively correlated with WC, TG, free testosterone, and FAI. In lean women with PCOS, the HOMA-M_120_ and Stumvoll index were significantly correlated with TG, free testosterone, FAI, and SHBG (all *P*s < 0.05) (Table 4).

**Table 4:** Correlation analysis between insulin resistance indices and metabolic and hormonal parameters in women with polycystic ovary syndrome.

Total PCOS (n = 100)	HOMA-IR		HOMA-M_120_		ISI-Stumvoll	
	**Correlation coefficient**	***P*-value**	**Correlation coefficient**	***P*-value**	**Correlation coefficient**	***P*-value**
**Waist circumference**	0.267	0.007	0.272	0.006	-0.289	0.004
**Triglycerides**	0.370	<0.001	0.439	<0.001	-0.480	<0.001
**HDL cholesterol**	-0.313	0.002	-0.319	0.001	0.361	<0.001
**Total testosterone**	-0.030	0.770	0.021	0.835	-0.013	0.897
**SHBG**	-0.310	0.002	-0.385	<0.001	0.405	<0.001
**Free testosterone**	0.319	0.001	0.408	<0.001	-0.436	<0.001
**Free androgen index**	0.343	<0.001	0.405	<0.001	-0.433	<0.001
**Lean PCOS (n = 50)**	**Correlation coefficient**	***P*-value**	**Correlation coefficient**	***P*-value**	**Correlation coefficient**	***P*-value**
**Waist circumference**	0.187	0.193	0.086	0.553	-0.030	0,839
**Triglycerides**	0.286	0.044	0.403	0.004	-0.418	0.003
**HDL cholesterol**	-0.159	0.269	-0.066	0.649	0.100	0.488
**Total testosterone**	0.003	0.985	0.024	0.870	-0.114	0.429
**SHBG**	-0.271	0.057	-0.285	0.045	0.306	0.030
**Free testosterone**	0.304	0.032	0.316	0.026	-0.412	0.003
**Free androgen index**	0.270	0.058	0.337	0.017	-0.386	0.006

HOMA-IR, homeostasis model assessment of insulin resistance; ISI, insulin sensitivity index; HDL cholesterol, high-density lipoprotein cholesterol; SHBG, Sex hormone-binding globulin.

## Discussion

In our study, women with PCOS who had normal glucose tolerance showed higher IR than age-, BMI-, and β-cell function-matched controls, and these differences held for lean women with PCOS compared to matched controls. β-cell function was increased in all women with PCOS compared to matched controls; however, β-cell function was not increased in lean subjects when compared alone. As described previously [12], the HOMA-M_120_ could be a simple tool to assess IR and was correlated with hyperandrogenemia and hypertriglyceridemia in lean women with PCOS.

IR was detected in over half of women with PCOS using fasting glucose and insulin [13]. Because of the higher risk for IR with PCOS, the risk of IGT and T2DM is higher in women with PCOS than in the normal population. The risk of IGT is two- to three-fold greater in women with PCOS, and the rate of conversion to T2DM is rapid [14]. Chinese women with PCOS and normal glucose tolerance showed higher IR than controls using the euglycemic-hyperinsulinemic clamp [3]. In agreement with the result of a previous study, we found that women with PCOS and normal glucose tolerance showed higher IR than controls. Although it is well known that women with PCOS have increased IR [15], few studies have investigated IR in lean women with PCOS, which is controversial. One study showed that lean European women with PCOS did not show higher insulin resistance than lean healthy controls using the euglycemic-hyperinsulinemic clamp [16], while in another study lean white women with PCOS had significantly higher IR than lean controls [17]. Lean Chinese women with PCOS have also been shown to have higher IR than controls [3]. Ethnicity could affect the differences observed in IR in lean women with PCOS between these studies. In this study, lean women with PCOS and normal glucose tolerance showed higher IR than age- and BMI-matched controls. These data suggest that IR is a characteristic feature of PCOS, which is independent of glucose tolerance status and obesity.

There are many methods for evaluating IR. The glucose clamp technique is regarded as the best available standard for the measurement of insulin action. However, the cost and time demands of the glucose clamp have led to the development of simpler methods to measure IR, such as the HOMA calculation [18]. Among the several available insulin resistance indices, the HOMA-M_120_ index has provided a very simple measure of IR in lean white women with PCOS [4]. Here we demonstrate for the first time that the HOMA-M_120_ is a useful index for evaluating IR in Asian women with PCOS. Lean women with PCOS, even when β-cell function is matched, showed higher values for the HOMA-M_120,_ but not the HOMA-IR, compared to matched controls in our study. The HOMA-M_120_ index was obtained from the calculation equation including post-load 2-hour glucose and insulin, however, the HOMA-IR index was obtained from the calculation equation including fasting glucose and insulin. Further studies are needed to evaluate the mechanism of IR in lean women with PCOS.

Previously, IR was reported to be significantly related to free testosterone levels [19]. In the present study, IR indices were significantly correlated with hyperandrogenemia in lean women with PCOS. In addition, the HOMA-M_120_ was positively correlated with hypertriglyceridemia in lean women with PCOS but the HOMA-IR was not. Approximately 70% of women with PCOS have dyslipidemia [20], and hypertriglyceridemia is a characteristic metabolic abnormality of insulin resistant individuals [21]. Therefore, the HOMA- M_120_ could be a useful tool for predicting IR as well as hyperandrogenemia and hypertriglyceridemia, which are important characteristics of PCOS.

PCOS is associated with profound IR as well as defects in insulin secretion [22]. However, the results of β-cell function analyses in women with PCOS were equivocal. Early impaired β-cell function (insulinogenic index) was detected in both lean and overweight/obese Chinese women with PCOS. The mean HOMA-F values were significantly lower in Chinese overweight/obese women with PCOS than those of BMI-matched controls [6]. In contrast to the results for Chinese women, women with PCOS in present study showed increased β-cell function compared to age- and BMI-matched controls. The Chinese women with PCOS included 4 patients with IGT. The inclusion criteria may affect the differences of β-cell function in women with PCOS. Similarly, both lean and obese PCOS subjects demonstrated a higher level of β-cell function than healthy women in an adult European population [23]. In another European study, women with PCOS also showed higher insulin secretion to maintain normal glucose homeostasis than age-, BMI-, and IR-matched controls [5]. Moreover, women with PCOS and increased β-cell function showed higher IR than matched controls in the present study. β-cell function was increased when all women with PCOS were compared to matched controls, but not when lean subjects with PCOS alone were compared to matched controls. The association of increased β-cell function and IR was weak in lean women with PCOS. These data suggest that increased β-cell function might be a characteristic of overweight/obese women with PCOS.

This is the first study to evaluate IR between women with PCOS and β-cell function-matched controls. The strength of our study is the use of well-matched age and BMI controls, which enabled us to perform group comparisons without bias. However, there are several limitations to our study. Ethnicity and age affect the prevalence of IR in women with PCOS [24]. Therefore, the results of our study can only be applied to Asian women with PCOS. Additional research is necessary to determine the patterns in other age and ethnic groups. Second, due to the small sample size, a selection bias might affect the results of our study.

Women with PCOS and normal glucose tolerance showed higher IR than age-, BMI-, and β-cell function-matched controls. Therefore, early evaluation of IR, even in lean women with PCOS and normal glucose tolerance, might be needed. Our data indicate that the HOMA-M_120_ could be a simple and reliable tool to assess IR in lean women with PCOS.
